# Addressing overuse of health services in health systems: a critical interpretive synthesis

**DOI:** 10.1186/s12961-018-0325-x

**Published:** 2018-06-15

**Authors:** Moriah E. Ellen, Michael G. Wilson, Marcela Vélez, Ruth Shach, John N. Lavis, Jeremy M. Grimshaw, Kaelan A. Moat, Synthesis working group, Sarah Garner, Roberto Grilli, Justin Peffer, Kevin Samra, Joshua Shemer, Terry Sullivan

**Affiliations:** 1 Department of Health Systems Management, Guilford Glazer Faculty of Business and Management and Faculty of Health Sciences, Ben-Gurion University of the Negev, PO Box 653, 84105 Beer-Sheva, Israel; 2 Institute for Health Policy, Management and Evaluation, University of Toronto, 4th Floor, 155 College St, Toronto, ON M5T 3M6 Canada; 3 McMaster Health Forum, McMaster University, 1280 Main St. West, MML-417, Hamilton, ON L8S 4L6 Canada; 4 Department of Health Research Methods, Evidence and Impact, McMaster University, 1280 Main St. West, Hamilton, ON L8S 4K1 Canada; 5 Centre for Health Economics and Policy Analysis, McMaster University, 1280 Main St. West, Hamilton, ON L8S 4K1 Canada; 6 Health Policy PhD Program, McMaster University, 1280 Main St. West, Hamilton, ON L8S 4K1 Canada; 7 Faculty of Medicine, University of Antioquia, Cra. 51d #62-29, Medellín, Antioquia Colombia; 8 Brown School of Social Work, Washington University in St Louis, 1 Brookings Dr, St Louis, MO 63130 United States of America; 9 Department of Political Science, McMaster University, Hamilton, Canada; 10 Department of Global Health and Population, Harvard School of Public Health, Cambridge, MA United States of America; 11 Clinical Epidemiology Program, Ottawa Hospital Research Institute, Ottawa, Canada; 12 Department of Medicine, University of Ottawa, Ottawa, Canada

**Keywords:** Overuse of health services, Waste, Low-value care, Health policy

## Abstract

**Background:**

Health systems are increasingly focusing on the issue of ‘overuse’ of health services and how to address it. We developed a framework focused on (1) the rationale and context for health systems prioritising addressing overuse, (2) elements of a comprehensive process and approach to reduce overuse and (3) implementation considerations for addressing overuse.

**Methods:**

We conducted a critical interpretive synthesis informed by a stakeholder-engagement process. The synthesis identified relevant empirical and non-empirical articles about system-level overuse. Two reviewers independently screened records, assessed for inclusion and conceptually mapped included articles. From these, we selected a purposive sample, created structured summaries of key findings and thematically synthesised the results.

**Results:**

Our search identified 3545 references, from which we included 251. Most articles (76%; *n* = 192) were published within 5 years of conducting the review and addressed processes for addressing overuse (63%; *n* = 158) or political and health system context (60%; *n* = 151). Besides negative outcomes at the patient, system and global level, there were various contextual factors to addressing service overuse that seem to be key issue drivers. Processes for addressing overuse can be grouped into three elements comprising a comprehensive approach, including (1) approaches to identify overused health services, (2) stakeholder- or patient-led approaches and (3) government-led initiatives. Key implementation considerations include the need to develop ‘buy in’ from stakeholders and citizens.

**Conclusions:**

Health systems want to ensure the use of high-value services to keep citizens healthy and avoid harm. Our synthesis can be used by policy-makers, stakeholders and researchers to understand how the issue has been prioritised, what approaches have been used to address it and implementation considerations.

**Systematic review registration:**

PROSPERO CRD42014013204.

## Background

Globally, health systems face the challenge of maximising value for money spent by maintaining or improving healthcare quality and efficiency in the face of shrinking or slow-growing budgets [1, 2]. Challenges in this area can be thought of in relation to underuse of beneficial services, misuse of services that provide benefits in some contexts but not others, and overuse of services [3, 4]. Overuse, and to some extent misuse, is referred to in the literature by many terms such as ‘too much medicine’, ‘low-value care’ , ‘inappropriate use’ , ‘obsolescence’ or ‘unnecessary care’ [5–7], but generally refers to “*care that can lead to harm and consumes resources without adding value to patients*” [8].

Compared with efforts to increase use of appropriate health services, efforts to address overuse have been minimal. However, increasing attention is being paid to this issue. This is partially attributable to the combination of slow-growing budgets and recognition that the wasteful use of public resources resulting from overuse limits the resources that can be deployed to address underfunded parts of health systems. Various studies have established the magnitude of the issue, finding that approximately a third of all patients (between 20% and 33%, depending on the study), receive treatments or services that the evidence suggests are unnecessary, ineffective or potentially harmful [9–11].

There are many examples focused on identifying overuse (e.g. health technology reassessment) [12] and/or addressing overuse (e.g. Choosing Wisely) [8]. Yet, these initiatives to address overuse have been criticised as fragmented, not fully established, and not always applicable to real-world settings as implementation is difficult and evaluation results are challenging to attain [13, 14]. The limited progress towards addressing overuse is therefore perhaps unsurprising given the complex interplay of the rationale used across differing contexts for addressing overuse, the processes or approaches that could be used to address overuse and the many potential barriers to implementing such approaches. Therefore, the purpose of this study was to develop an in-depth understanding and framework of addressing overuse of health services in health systems. Our guiding objectives were framed based on key domains of policy development, focused on (1) the rationale and context for why overuse is prioritised on policy agendas as an issue to be addressed in health systems; (2) the processes and approaches, programmes and policies that can be or have been used to reduce overuse of health services in health systems; and (3) implementation considerations for addressing overuse at the system level.

## Methods

We used a critical interpretive synthesis (CIS) approach paired with an integrated knowledge translation approach that included a stakeholder engagement process (a stakeholder dialogue) allowing us to refine and triangulate our findings based on the views and experiences of policy-makers, stakeholders and researchers involved in addressing overuse. Given that our methods are detailed in a peer-reviewed published protocol [15], we focus here on providing an outline of the key methodological features of CIS and how the stakeholder dialogue contributed to shaping our findings. In brief, a CIS has the core objective of developing a theoretical framework using insights and interpretation drawn from a wide spectrum of relevant empirical and non-empirical sources. Given this, we chose CIS for our methodological approach because it is ideal for questions which draw on literature that is not well developed or focused [16, 17] (as is with this issue), and because of its focus on developing frameworks which draw from a broad range of relevant sources (i.e. not just those that meet particular design or quality criteria).

Our approach adopted methods from traditional systematic reviews (e.g. systematic and transparent database searches and having two reviewers independently review search results) paired with purposive sample and inductive analysis. This included (1) developing a compass question that can evolve (in a transparent way) over the course of the review; (2) iteratively revising inclusion/exclusion criteria to reflect changes in the compass questions; (3) purposively sampling literature outside of the original search parameters to fill conceptual gaps; (4) including empirical and non-empirical documents that offer unique insights; (5) purposively sampling from the eligible articles to focus the analysis on those with the most relevance and insights; and (6) using an iterative approach to analysis of the extracted information from purposive sample articles to derive a framework. Our process therefore began with a broad idea of developing a framework and our process for doing so evolved according to themes identified from the literature and from our stakeholder engagement process.

### CIS methods

We employed both an explicit and structured approach to searching and reviewing the indexed literature (similar to traditional systematic reviews) and an inductive approach that is typically associated with CIS. This ensured that the final sample of included papers was theoretically rich and relevant to our objectives. Specifically, we searched (in July 2014 and again in May 2015) 15 databases that index research literature on a diversity of subject domains. The detailed search strategy (Additional file 1) included terms used to describe overuse and efforts to address it (e.g. disinvestment, obsolescence, overuse), contextual terms (e.g. rationing, resource allocation) and limiting terms (e.g. health care, health). These searches were supplemented by a targeted search for literature about Choosing Wisely campaigns, and searches conducted to inform the development of an evidence brief to inform the stakeholder dialogue that we convened (see description below).

Two reviewers independently screened the retrieved titles and abstracts of all articles written in English or Spanish (languages spoken by the team) that focused on addressing overuse at a macro (i.e. national and sub-national) or meso (i.e. regions, organisations or networks) but not micro level (i.e. individual clinicians or teams of clinicians). Two reviewers then assessed the full text of any articles identified as ‘potentially relevant’. Articles were included in the final sample if they were deemed to provide clear insights into one or more of our three objectives. The included articles were conceptually mapped by one reviewer and checked for consistency by another. Any disagreements in categorisation between the reviewers were resolved before proceeding. Each article was identified as being relevant to at least one of our core areas of interest (i.e. context and rationale, processes and implementation) and mapped using frameworks relevant to these areas, which included (1) government agenda-setting (through Kingdon’s framework) [18]; (2) policy development (through the 3I + E framework derived from political science and relating to institutions, interests, ideas and external factors that affect policy-making) [19]; and (3) implementation and health system arrangements (through the Health Systems Evidence taxonomy of governance, financial, and delivery arrangements, and implementation strategies) [20]. The specific categories included in each of these frameworks are described in detail in our published protocol [15].

The project leads (MEE and MGW) used conceptual mapping to select a purposive sample of articles for the final analysis. Our selection of the final sample followed a two-stage approach. Firstly, we included all systematic reviews and articles that we deemed to be conceptually rich, defined as having relevance to two or more of our areas of interest and to two or more of our analytical frameworks (i.e. those that spanned multiple research questions and the policy process stages). Additionally, we purposefully sampled three to five of the most conceptually rich articles from approaches that were the focus in a large number of articles (e.g. Choosing Wisely). Second, we reviewed the full text of remaining articles and included those that (1) explored a process or approach that were not yet captured in the first stage; (2) provided a complementary perspective to an approach or process that was already included; (3) captured a breadth of perspectives across different countries; (4) described evaluations of processes designed to address overuse; (5) integrated many different concepts into one manuscript; and (6) provided perspectives from disciplines outside of health. We prioritised the inclusion of empirical articles whenever possible.

Data extraction for articles included in the purposive sample was conducted by writing a 1–2 paragraph summary of key messages, documenting article characteristics and extracting findings according to variables included in the same frameworks we used to conceptually map articles. We used a constant comparative method for data analysis, which ensured the framework development drew from both the data provided from the research and the collective interdisciplinary skills and experience of the study team.

### Stakeholder engagement

We supplemented our CIS with a stakeholder-engagement process to refine our understanding of themes emerging from the synthesis and support action from health-system leaders in Canada on the issue. Specifically, we convened a stakeholder dialogue with Canadian policy-makers, stakeholders and researchers who were purposefully selected by the research team based on their ability to bring unique insights to the issues and their ability to champion change following the dialogue (Wilson et al., Addressing Overuse of Health Services in Canada: Findings from a stakeholder dialogue with Canadian health-system leaders, submitted; [21]). In preparation for the dialogue, we developed an evidence brief that mobilised local and global evidence drawn from (1) our ongoing CIS, (2) searches for evidence relevant to the Canadian context, as well as systematic reviews relevant to the policy options included in the brief, and (3) insight from key informant interviews conducted with health system leaders and researchers in Canada and internationally to refine and triangulate our findings from the CIS.

For the stakeholder dialogue, we convened 19 participants, namely seven policy-makers, two managers, one healthcare professional, five researchers, and four representatives from national health system organisations (one participant from the United States and all others from Canada). The deliberations were thematically summarised in relation to the problem, elements of an approach to address the problem, implementation considerations and next steps. We used the results from the evidence brief and dialogue (Wilson et al., Addressing Overuse of Health Services in Canada: Findings from a stakeholder dialogue with Canadian health-system leaders, submitted; [22]) to triangulate the core constructs of our framework and to further understand contributing factors.

## Results

**Fig. 1:**
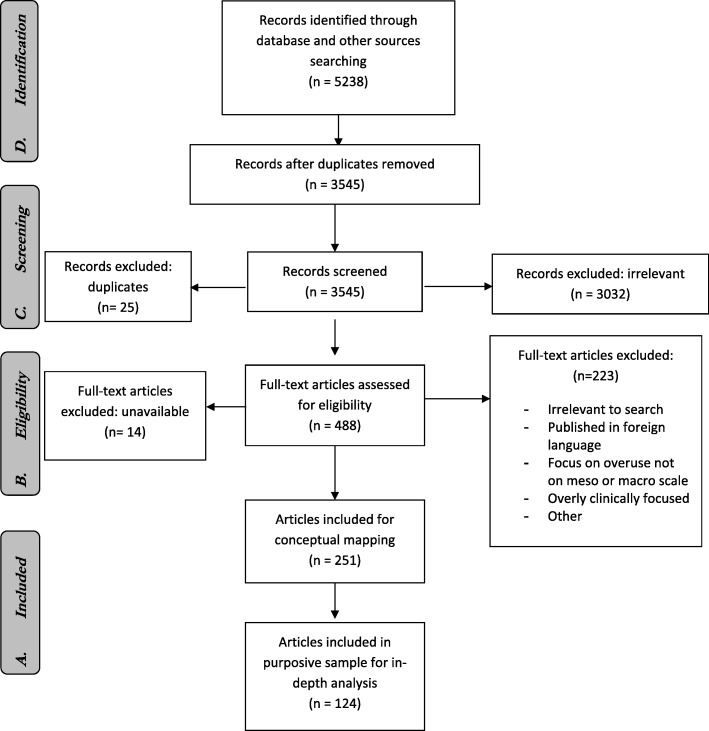
PRISMA flow diagram

After duplicate records were removed, we screened 3545 references for eligibility. We excluded 3057 records due to lack of relevance and duplicates, leaving 488 potentially relevant articles (Fig. 1). After full-text review, we included 251 for conceptual mapping, from which we selected a purposive sample of 124 articles. In Additional file 2, we provide a list of the articles excluded during full-text review, those included for conceptual mapping, those included and excluded from the purposive sampling and the characteristics of included articles.

Most articles included in the conceptual mapping (76%; *n* = 192) were published between 2010 and 2015 (Table 1). The majority of articles addressed a process for addressing overuse (i.e. programmes and policies) (63%; *n* = 158) or political and health system context (60%; *n* = 151), followed by agenda-setting and prioritisation (27%; *n* = 67), implementation considerations (25%; *n* = 63), rationale (24%; *n* = 61) and policy development (17.5%; *n* = 44). Nearly all articles (94%; *n* = 235) had a specific country or region of focus, and usually only one country in each article (85%; *n* = 213), although a few had global focus (6%; *n* = 16). Additionally, nearly all articles focused on high-income countries (98%; *n* = 245).

**Table 1:** Number of studies addressing main themes based on different variables

	Rationale and Context (*n* = 179)	Process (*n* = 158)	Implementation (63)
Years published	1990–1999 = 12 2000–2009 = 31 2010–2016 = 136	1990–1999 = 9 2000–2009 = 27 2010–2016 = 122	1990–1999 = 1 2000–2009 = 10 2010–2016 = 52
Study type	Research = 78 Non-research = 101 Systematic review = 13 Cross sectional = 12 Qualitative study = 21 Case study = 17 Mixed methods = 9 Cohort study = 1 Interrupted time series = 1 Other research = 4	Research = 90 Non-research = 68 Systematic review = 14 Cross sectional = 12 Qualitative study = 17 Case study = 25 Mixed methods = 9 Cohort study = 1 Interrupted time series = 1 Before and after = 1 Other research = 8	Research = 45 Non-research = 18 Systematic review = 8 Cross sectional = 5 Qualitative study = 7 Case study = 12 Mixed methods = 3 Interrupted time series = 4 Before and after = 1 Other research = 5
Country focus	Australia = 33 Canada = 28 United Kingdom = 57 United States of America = 66 Spain = 14 Netherlands = 4 New Zealand = 4 Italy = 6 France = 5 Germany = 5 Ireland = 2 Denmark, Japan, Sweden, Switzerland, Austria, South Korea = 1	Australia = 19 Canada = 33 United Kingdom = 41 United States of America = 68 Spain = 11 Netherlands = 2 New Zealand = 6 Italy = 5 France = 2 Germany = 2 Ireland = 1 Norway, Denmark, Japan, Sweden, Switzerland, Austria, South Korea = 1	Australia = 8 Canada = 19 United Kingdom = 20 United States of America = 20 Spain = 3 Netherlands = 1 New Zealand = 3 Italy = 4 Israel = 2 Sweden = 3 South Korea = 2 France = 1 Switzerland = 1

### Framework for addressing overuse of health services

**Fig. 2:**
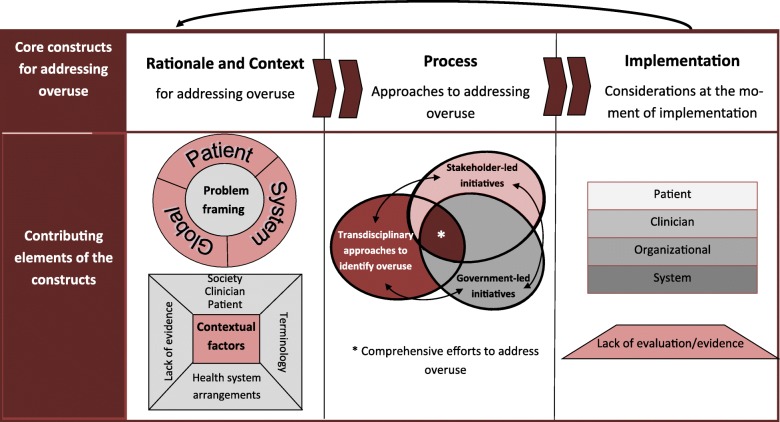
Framework for understanding and addressing the issue of health services overuse

The core constructs of our framework were supported by our analysis and through the stakeholder engagement process. We depict the core elements of the framework in Fig. 2 and describe the contributing factors related to each of the three constructs below.

### Rationale and context for why overuse is prioritised to be addressed in health systems

Issues are typically prioritised for attention on government agendas through the emergence of a compelling problem or political factors [23]. A variety of framings of the problem contributed to overuse gaining increased attention, which is driven by a range of contextual factors. We found that the problem is framed in relation to negative outcomes at the patient, system and global level. At the patient level, the problem of overuse can manifest as patient harms and/or low-quality care. For example, in the area of prescription medications, despite studies demonstrating high risks associated with prolonged use, there is substantial overuse of benzodiazepines among older adults, which can lead to higher rates of motor vehicle accidents, falls and hip fractures, potentially leading to hospitalisation and death [23, 24]. In addition, the use of low-value tests in low-risk populations can lead to false-positive results, and further unnecessary investigations exposing patients to harms such as side effects or interactions with other medications.

When magnified at the system level, overuse accounts for up to a third of patients receiving ineffective or harmful care [9–11]. This contributes to significant system-level fiscal constraints that can limit the scope of policy levers that policy-makers can use to strengthen health systems. In particular, they may have to forgo investments in underfunded parts of the system that could provide beneficial services and drugs [25]. Lastly, overuse can lead to far-reaching negative outcomes at the global level (e.g. antibiotic overuse leading to antimicrobial resistance, which affects every country) [26].

We also identified four groupings of contextual factors that drive the issue, namely (1) culture at the societal, clinician and patient levels; (2) lack of agreement on framing or terminology to describe the issue; (3) health-system arrangements; and (4) a lack of evidence regarding optimal approaches to addressing overuse.

In Table 2, we outline the societal, clinician and patient factors, all of which can play a role in contributing to overuse of health services. At the societal level, the ideas that ‘more is better’ [27] and ‘new is better’ [28] are further entrenched by overall market forces creating increased demand for products, and which often exist without any counteracting force [29, 30]. At the clinician level, there is a prevailing culture of thoroughness [31, 32], fear of litigation and accusation of medical malpractice, fuelling overuse [28, 32]. There can also be a lack of awareness by some providers that they overuse health services, and blame-shifting to providers working in different specialties [31]. Underpinning these factors are the difficulties encountered when changing established ways of providing care [28, 33]. Patient-level factors are also intertwined with the prevailing societal culture of ‘more is better’ and they may demand unnecessary treatments, which may be supported by poor health literacy and/or opportunities for meaningful engagement in their care to make informed judgments [9, 32]. Perhaps as a corollary, there can be a feeling that providers are ‘better’ if they do more [31].

**Table 2:** Societal, clinician, and patient-level considerations affecting overuse of health services (table adapted from Ellen et al.) [21]

Level	Considerations	Explanation
Societal	• Culture of ‘more is better’	• The idea that ‘more is better’ permeates all aspects of society, including healthcare, which contributes to clinicians and patients to often opt for more tests or procedures, or take more drugs
	• Market forces	• Market forces that create increased demand for products and often exist without a counteracting force that makes the case for why more is not always better
Clinician	• ‘Better safe than sorry’ approach to care	• There is a prevailing culture of thoroughness and ‘better safe than sorry,’ which can mean ordering unnecessary tests ‘just to be sure’
	• Acknowledgement of the issue and blame avoidance	• Some specialties have difficulty acknowledging that a service, test or procedure in which they have a vested interest may be overused • When provider groups have been asked to create lists of low-value services, they tend to include recommendations for other clinicians about what to do (or not to do) rather than address overuse by themselves and their colleagues
	• Ability or willingness to change established ways of providing care	• As established in the behaviour-change literature, changing the way clinicians practice can be difficult and takes time
Patient	• Perception that clinicians that do more are better	• Receiving a test or treatment, even if it does not offer measurable benefits, is often seen as needed as the logical end point of an interaction between a patient and clinician
	• Demand for tests (e.g. from ‘well-informed’ patients) that are not evidence based	• Patients may not believe that their particular service, test or procedure is of low value and, when they are ill, disregard efforts to address overuse that are ‘for the greater good’ • While the information presented by patients to their clinician may be accurate, they may not be fully informed about what they need and hence many demand too many services and/or services that are inappropriate
	• Citizen/patient health literacy	• Limited health literacy is a barrier to understanding health information and necessary alternatives, which can lead to the overuse of health services such as emergency room visits and hospitalisations
	• Patients are not always consulted in decision-making processes	• Patients are often not engaged, or are engaged too late in the process and, as a result, do not fully understand, appreciate or agree with the decisions being proposed by their provider

The framing of efforts to address overuse also contributes to the problem and can lead to inertia among groups needing to take action. For example, the language of discontinuing and disinvesting, and the associated cost savings implied by these terms, may resonate with organisations and governments. In contrast, clinicians may react negatively to these terms believing infringement on their autonomy and ability to determine appropriateness of care for their patients. Patients may also equate these terms with their care being rationed, even when a service is replaced by a new one of similar value [34]. Finally, depending on the political culture, politicians may want to avoid using these terms given the potential to be seen as reducing benefits for their constituents.

As outlined in Table 3, our analysis also points to overuse being driven by a complex interplay of system-level factors. At the delivery level, limited training or preparation of clinicians to addressing overuse [8], the minimal time that clinicians have with patients [35], a lack of awareness of recent evidence, fragmented delivery of services across the system that contributes to inefficient delivery of care [36] and co-dependence of service delivery, contribute to the problem [37]. Similarly, many aspects of financial arrangements in health systems, such as financing systems, remunerating providers [31], and purchasing products and services [38], can drive overuse. Governance arrangements can contribute to overuse when there is a lack of role clarity, such as when physicians do not see themselves as resource stewards and therefore do not consider or discuss the financial implications of ordering various services with patients [39].

**Table 3:** System-level arrangements that contribute to overuse of health services

Type of system arrangement	Factor	Explanation
Delivery	• Limited training or preparation of clinicians to contribute to addressing overuse	• Most healthcare providers currently do not have the necessary skills to have the conversation regarding procedures that may be unnecessary or harmful to the patient
	• Limited time with patients	• Healthcare providers continually state that time constraints with patients is a barrier for practicing shared decision-making and explaining the reasoning behind not ordering specific health services
	• Fragmented delivery of services across the system	• Patient information may not be shared effectively across providers, leading to duplicate and unnecessary testing for patients leading to inefficient care
	• Co-dependency of service delivery	• It is challenging to withdraw resources from one health service without affecting others which are supplemental or dependent on the service being withdrawn from
Financial	• Financing systems	• Many health insurance cost-sharing approaches are applied to all services, regardless of clinical benefit • Identifying the correct balance within cost-sharing is difficult • High out-of-pocket spending may reduce the use of high-value services, while low out-of-pocket spending may lead to the overuse of unnecessary services
	• Remunerating clinicians	• Fee-for-service remuneration incentivises the provision of services, regardless of their value, and providers may be reluctant to reduce their use as their income will be negatively affected • Physicians also lack incentives to ration services
	• Purchasing products and services	• The use of financial ‘levers’ to address overuse is only helpful in specific contexts (e.g. withholding funding for specific health services that are harmful), and are far too simple to be used to address the overuse of services that may provide minimal or no benefit for certain subgroups, but that may benefit others, or that may be more expensive or cost-ineffective, but are valued by some patient subgroups
Governance	• Role clarity in the system	• Many providers do not see themselves as resource stewards and therefore often do not consider or discuss the financial implications of ordering various tests, treatments and procedures with patients
	• Tension between autonomy and accountability	• While clinicians and organisations are given autonomy to decide which services are necessary, there is also a need for accountability measures to be put into place to enforce appropriate use
	• Stewardship and authority	• Overlapping authority on different governmental levels make withdrawing from services difficult • Leadership to tackle the issue may be lacking

Finally, while numerous initiatives exist to address overuse (as outlined in the next section), there has been criticism that they are fragmented [14, 40, 41] and that the evidence available to inform decisions is minimally helpful or non-existent [42–44]. This has pointed to a need to address the issue in real-world settings, as opposed to controlled, purely academic studies. Additionally, initiatives to address overuse have not been well evaluated, with much of the literature emphasising that implementing them is difficult and that the intended impacts of reducing overuse are hard to achieve [40, 41, 45].

### Processes and approaches to addressing overuse

We identified three elements of a potentially comprehensive approach to address the issue of overuse of health services, namely (1) transdisciplinary approaches to identify health services that are overused, (2) health-system stakeholder-led initiatives to address overuse and (3) government-led initiatives to address overuse. While these three processes are presented separately below, a comprehensive approach (Fig. 2) likely lies in synergistic efforts between stakeholders and governments to identify overuse and subsequently undertake collaborative efforts to address it.

In Table 4, we provide an overview of each element, the processes/components that the elements could include and examples. While it was beyond the scope of this synthesis to provide a comprehensive review of the evidence about the benefits, harms and costs of these processes, since many are comprised of large bodies of research, we provide a high-level overview of the evidence in our evidence brief [22] and in a separate publication (Wilson et al., Addressing Overuse of Health Services in Canada: Findings from a stakeholder dialogue with Canadian health-system leaders, submitted).

**Table 4:** Processes to address overuse

Elements	What it could include	Examples
Transdisciplinary approaches to identify health services that are overused	• Use the best available data, research evidence and guidelines to identify overuse of health services	Different approaches exist to use existing evidence to identify areas of overuse; for example, in the United Kingdom, NICE creates ‘do not do’ recommendations. This is done by advisory bodies using health technology assessments to identify areas of practice that are ineffective or lack sufficient evidence to support their continued use [76]
	• Conduct jurisdictional scans to identify health services that have been delisted due to overuse in other health systems using evidence-based processes and determine whether the same services are still being used locally	Various examples of conducting jurisdictional scans were reported in the literature, e.g. the EuroScan network is a collaborative, global network which conducts healthcare horizon scanning for health technologies. Its use revealed that approximately a quarter of technologies introduced into the health systems surveyed could be removed, as they are substitutes for already existing technologies [77]
	• Identify health services that should be prioritised for full or partial removal from the health system through stakeholder- and consumer-engagement processes	Stakeholders can be engaged in a multitude of ways; for example, across seven programmes for identifying ineffective health technologies at different levels, stakeholders were involved in all programmes, either as consultants, or as part of an advisory panel or working group [78]
Stakeholder-led initiatives to address overuse	• Foster better communication and shared decision-making between providers and patients based on evidence-based recommendations and best practices	One example of an initiative to increase communication and shared decision-making is the Choosing Wisely campaign, which develops lists of “*things patients and physicians should question*”; by using this terminology instead of stating what not to do, respectful dialogue about informed choice-making is promoted between physicians and patients [79]
	• Change provider behaviour to address inappropriate use of health services in their practice	Different interventions have been developed to change provider behaviour such as guidelines, training sessions for primary care physicians, cost displays and order form changes [80]
	• Educate patients/citizens about what health services they need (e.g. through decision aids)	Educating the users can assist in reducing overuse; for example, educating consumers can reduce the overuse of unnecessary and at times harmful medications [81]
	• Develop mass-media campaigns to raise awareness about the need to address overuse	Mass media campaigns that target the public and increase awareness can be used effectively; for example, a mass media campaign to reduce the use of antibiotics reduced retail pharmacy antibiotics by 3.8% and managed care-associated antibiotic dispenses by 8.8% [82]
Government-led initiatives to address overuse	• Revise lists of publicly financed products and services	Revising lists of publicly funded services to remove services that are of low-value; for example, in Spain, low-value technologies are limited by level of reimbursement, frequency of use or restriction by patient or provider type [83]
	• Modify remuneration for providers or incentivise consumers to prioritise the use of some products and services over others	Incentives with outcome monitoring on the supply side reduce the use of low-value care through partial capitation or shared savings; value-based insurance design is one of the processes used to increase co-pay on services that are likely to be unnecessary [84]
	• Require prior authorisation for use of specific health services that are identified on a list of overused services	Both demand and supply-side interventions need to make using low-value health services financially unwise or difficult to use without authorisation. This includes service specific interventions such as pay for performance, prior authorisation and population-based interventions, such as risk sharing, where providers accept financial responsibility for total costs of care [27]
	• Engage stakeholders and consumers in decision-making processes	Giving stakeholders a voice will ultimately enforce their support for whatever result is reached, even if it was not their preferred one [85]. Relevant methods include consensus techniques, coverage design, integrating the evidence from systematic reviews with social values and preferences, and prospective data collection [41]

Transdisciplinary approaches to identify overused health services could draw on processes to (1) use the best available data, research evidence and guidelines; (2) conduct jurisdictional scans to identify services that have been overused in other health systems, and determine whether those services are being used locally; and (3) engage stakeholders and consumers to identify services that should be prioritised for full or partial de-implementation. We identified several examples, including NICE’s “*do not do*” recommendations [42]; the use of systematic reviews (e.g. Cochrane reviews) [6] or practice variation studies [46, 47]; conducting health technology assessments (or reassessments) [12, 48]; and programme budgeting and marginal analysis [49–51].

Once areas of overuse have been identified, two different approaches can be pursued separately or in tandem. The first approach is focused on stakeholder-led/bottom-up initiatives (many of which could be implemented in collaboration with governments) drawing on processes for (1) fostering better communication and shared decision-making between providers and patients based on evidence-based recommendations [52, 53]; (2) changing provider behaviour to address overuse [54–57]; (3) educating patients/citizens about needed health services [58–60]; and (4) developing mass-media campaigns to raise awareness on the issue [61–63]. The Choosing Wisely campaign, which targets clinicians, patients and other stakeholders in an effort to raise awareness about and support behaviour change to address overuse, is a notable example of a stakeholder-led approach given its prominence in the literature and because it has adopted several of these processes [8]. Yet, the evidence about the impact of Choosing Wisely and other initiatives is limited.

Supporting behaviour change among clinicians is also important (Wilson et al., Addressing Overuse of Health Services in Canada: Findings from a stakeholder dialogue with Canadian health-system leaders, submitted; [22]). Such processes must first identify or diagnose the behaviours that need to change to address overuse through systematic/structured [64] or iterative/theory-based approaches (e.g. by using the Behaviour Change Wheel) [65, 66]. This step is essential to selecting the types of strategies that can support behaviour change and iteratively refine and tailor them to ensure they are combined to maximise impact [67].

The second approach, government-led/top-down, could draw on four types of processes, namely (1) revising lists of publicly financed products and services; (2) modifying remuneration for providers or incentivising consumers to prioritise the use of some products and services over others; (3) requiring prior authorisation for use of specific health services that are identified on a list of overused services; and (4) engaging stakeholders and consumers in decision-making processes. As with the stakeholder-led approaches, these could similarly be developed and implemented in collaboration with stakeholders as part of a comprehensive approach.

### Implementation considerations

Numerous barriers were identified at the patient/citizen, clinician, organisation and health-system levels (Table 5). These include barriers at the level of the patient (e.g. the constant access to a plethora of information can be confusing and may fuel demand for unnecessary services) [68], citizens (e.g. they may view such approaches as rationing services, which may be politically unpalatable) [34], clinicians (e.g. they may resist interventions that they view as an encroachment on their autonomy and ability to generate income) [28, 69, 70], organisation (e.g. they may have competing interests, limiting willingness and/or ability to engage in collaborative approaches) [40] and systems (e.g. a lack of stakeholder awareness or political will) [34, 71].

**Table 5:** Implementation barriers

Categories	Barriers	Explanation
Patient/public level	• Online information	• Patients have access to websites and advertisements of varying quality that may lead them to asking for tests or procedures that they do not need
	• Resistance: not consulted early enough	• The shared decision-making process needs to be perceived as legitimate and transparent, especially by the public; however, the level of public involvement may vary, depending on the nature of the decision and the personal preferences of members of the public
	• Resistance: may not want services rationed	• The natural inclination of people (patients and clinicians alike) is to perceive a greater disadvantage from the withdrawal of an already existing service as opposed to the denial of a new service of similar value; additionally, patients may feel entitled to services that have been available in the past
	• Funded by interest groups	• Some patient groups may be funded by the manufacturers of drugs and technologies, and these groups could influence stakeholder- and consumer engagement processes to identify overused health services
	• Lack of information	• Some patients may not feel sufficiently informed to properly contribute to the shared decision-making process
Clinician	• Obtaining agreement from providers regarding what is unnecessary	• Some providers may not be aware of or agree with the services that have been identified as overused and they may view the service as necessary, which could be the result of many reasons such as publication bias (i.e. where they read mostly what should be done and not what should not) or industry pressure where more is viewed as better
	• Providers may resist the encroachment on autonomy and income	• Providers, even if they do understand about overuse and its implications, may still be reluctant to accept limitations on their service use as this goes against their financial incentives, as well as against patient choice and provider autonomy to decide which treatment options are best
	• Providers may view this as a passing fad	• Providers may perceive these initiatives as just another passing fad, and therefore may not invest energy in them
	• May focus on low hanging fruit or other specialties	• Providers may not want to withdraw funds from services within their specialty, and therefore will only focus on low-hanging fruit or shift responsibility to other specialties and their overuse
Organisation	• May have competing interests	• Some organisations may have competing interests and priorities and therefore may resist collaborating with such an initiative
	• May need extra resources	• Organisations may view such an initiative as requiring extra organisational resources (e.g. shared decision-making requires more time with patients and hence more resources)
	• Fatigue due to too many initiatives	• Some organisations may be experiencing fatigue (e.g. some organisations and their management may be tired of new ideas so there may be resistance to implementing another new initiative)
	• Lack of infrastructure	• Some organisations may not have the infrastructure to implement the necessary changes
Health system	• Lack of awareness	• Some health system leaders may not be aware of the issues and the potential negative outcomes of the overuse of health services
	• Lack of political will	• Some health system leaders may lack the political will to address the overuse of health services
	• Coordination between levels in the health systems	• Building consensus between stakeholders and different levels of government will be challenging, which will make coordination at a national level challenging
Lack of evaluation/evidence	• Lack of evidence that certain tests/treatments are being overused	• There is a lack of evidence and published literature that clearly demonstrate that existing health services provide little or no benefit, and at times cause harm • The data to demonstrate that there is overuse does not always exist or is not always easily accessible
	• Lack of information that certain organisations or providers are actually overusing the service	• Stronger evidence and access to data is needed to demonstrate overuse across providers, organisations and jurisdictions
	• Lack of belief that the current evidence is correct (i.e. when physicians are presented with variations of care, they question the evidence)	• When presented with evidence of overuse, many providers challenge the evidence base and are resistant to accept the results

### Insights from the stakeholder dialogue

As stated above, our framework is based not only on findings from the literature, but also from a stakeholder dialogue convened with 19 health-system leaders in Canada, which we report on in detail elsewhere (Wilson et al., Addressing Overuse of Health Services in Canada: Findings from a stakeholder dialogue with Canadian health-system leaders, submitted; [22]). It is worth noting that dialogue participants emphasised particular components of the framework. For example, in relation to the challenges associated with addressing overuse, participants (which were provided a rationale and context for addressing it) emphasised the lack of common terminology that prevents a shared understanding of the issue, the complex and interrelated causes of overuse which make it challenging to comprehensively address, as well as differing political and system contexts that further complicate coordinated efforts to address overuse across Canada. In deliberating about approaches to address overuse, participants consistently identified the need for leaders to take ownership of the issues and to coordinate comprehensive efforts that include transdisciplinary approaches to identify and diagnose overuse combined with both stakeholder- and government-led efforts to address it. Finally, in terms of moving forward with actions to address overuse, participants emphasised the need for enhanced efforts to collect and share data and fostering synergistic efforts in ways to build a sustained commitment among stakeholders and policy-makers to address overuse.

## Discussion

Addressing the overuse of health services is a health-system issue that has gained increasing traction with policy-makers and stakeholders over the past decade. As our CIS emphasises, health service overuse is a complex issue. Numerous initiatives have been identified in different countries focusing on identifying and reducing common areas of overuse, yet they have not been well evaluated. Implementation is difficult and results are hard to identify and achieve. While numerous barriers exist, the biggest barrier may be the complex interplay between the societal culture of ‘more is better, ’ the competing priorities among different actors in the system, and the willingness of policy-makers to make politically difficult decisions.

This synthesis makes a significant contribution to the knowledge base on overuse and expands upon other work on the issue. Other reviews have had somewhat of a narrower focus (e.g. focusing on one element of the broader issue), while our synthesis tackles the broader issue of overuse [43, 72, 73]. Furthermore, while there are recent overviews of drivers of overuse, policy approaches and change levers which may address overuse [74, 75], our process was more comprehensive and grounded in an approach that paired a thorough and robust synthesis with a stakeholder-engagement process.

Several key implications can be derived from our analysis. Firstly, while addressing overuse is complex, there is much interest in its many components, including reducing patient harm and improving quality of care and health system efficiency. Given this, it seems to be an opportune time for health system stakeholders to capitalise on this interest and comprehensively address overuse. Secondly, addressing overuse will likely be most successful by combining transdisciplinary, systematic and transparent aspects to determine what is driving overuse and coordinating efforts from stakeholders and governments to address overuse. The replicative nature of current initiatives, originating from different parts of the health system but having the same overall goal, is redundant. A comprehensive, system-wide approach that is coordinated between relevant stakeholders should be the norm. Such synergistic efforts were emphasised during the stakeholder dialogue (Wilson et al., Addressing Overuse of Health Services in Canada: Findings from a stakeholder dialogue with Canadian health-system leaders, submitted; [22]). While our findings indicate that numerous initiatives have been implemented, there are limited evaluations of their impact, making it difficult to determine what works in different health and political system contexts. As such, more attention needs to be given to studying the impact of approaches to addressing overuse. This likely requires a commitment of policy-makers to ensure monitoring and evaluation is included as part of any system-level responses. Such commitment and investment will not only support efforts to address overuse, but will make possible policy responses that can be continually refined and optimised over time.

Finally, given that decisions to address overuse ultimately affect the types and quality of care received by patients, it will be important to engage them to address overuse. This could include greater involvement in clinical decision-making or broader citizen engagement to identify values and preferences for directions to take at a system level.

A key strength of this synthesis is that we used methodologies similar to those employed by systematic reviews to identify and select the articles, as well as purposive sampling of the literature to fill conceptual gaps and an iterative approach to the analysis. Thus, our analysis included insights from both empirical and non-empirical literature, which often provides helpful insights for complex policy questions. Furthermore, pairing our synthesis with stakeholder engagement allowed us to ‘road test’ and triangulate many concepts.

Some potential limitations are worth noting. As this is not a traditional systematic review but resembles more of a qualitative analysis, it could be viewed by some as being a subjective rather than objective assessment of the literature. However, we ensured that our process was systematic, transparent, robust and aligned with other examples of CISs by having at least two researchers independently conduct each stage and validating our findings through key informant interviews. Another potential limitation is that, given the prominence of overuse in the literature in the last several years, new articles are constantly being published (although this is not unique to our review). We attempted to address this by monitoring key sources for literature (e.g. through relevant listservs), updating our search, and including findings from the evidence brief that we produced after the final set of searches.

## Conclusion

Our work can be utilised by policy-makers, stakeholders and researchers to gain insight about the prioritisation of overuse and the structure and implementation of initiatives used to address it. These approaches can support stakeholders to address overuse and improve health system effectiveness and efficiency. Moreover, our findings point to the need to foster comprehensive approaches to address overuse, including coordinated efforts among many stakeholders (including citizens and patients) to identify and determine what is driving overuse, identify the types of actions that are needed to address it, and evaluate the impacts of such approaches. Without such comprehensive approaches, overuse of health services will remain a significant issue and continue to strain health systems and limit policy-makers’ ability to invest in areas that remain underserved.

## Abbreviation

CIS: Critical interpretive synthesis
